# Acceptance of Illness, Quality of Sleep and Emotional State of Adolescents with Lymphatic Malignancy During the First Cycle of Anticancer Treatment—A Preliminary Report

**DOI:** 10.3390/healthcare13060637

**Published:** 2025-03-14

**Authors:** Agnieszka Kruszecka-Krówka, Grażyna Cepuch, Anna Królikowska, Agnieszka Micek

**Affiliations:** 1 Nursing and Midwifery Institute, Faculty of Health Sciences, Jagiellonian University Medical College, 31-501 Krakow, Poland; grazyna.cepuch@uj.edu.pl; 2 Department of Pediatric Oncology and Hematology, University Children’s Hospital of Krakow, 30-663 Krakow, Poland; krolikowska_ania@o2.pl; 3 Statistical Laboratory, Faculty of Health Sciences, Jagiellonian University Medical College, 31-501 Krakow, Poland; agnieszka.micek@uj.edu.pl

**Keywords:** lymphomas, adolescents, anxiety, depression, irritability, sleep disorders, pain, acceptance of illness

## Abstract

**Background**: Medical care provided to adolescents with lymphatic system cancer and leukemia should take into consideration psychological aspects, due to the increased risk of anxiety disorders, depression, irritability and sleep disorders, which may determine acceptance of the disease. **Methods**: The study included 50 patients of both sexes, aged 14–17. The following questionnaires were used: The Hospital Anxiety and Depression Scale (HADS-M), Athens Insomnia Scale (AIS), Acceptance of Illness Scale (AIS) and Numeric Rating Scale (NRS). **Results**: A significant group of patients did not accept their disease. Male gender turned out to be a predictor of higher acceptance of the disease. The dominant emotion among young people was anxiety, especially among girls (*p* = 0.012). The level of depression of most respondents was low or moderate (*p* = 0.143), and irritability was high (*p* = 0.074), regardless of gender. Sleep disorders were more common in girls (*p* < 0.001) and were associated with high levels of anxiety and depression. Regardless of gender, most adolescents experienced pain (≥3 NRS). **Conclusions**: Recognizing the predictors of disease acceptance, especially in the initial stage of treatment, may be of key importance for current and further therapeutic effects in adolescents; therefore, it should be included in the standards of care for this group of patients.

## 1. Introduction

B-cell lymphomas belong to the most common forms of cancer in children and adolescents and can be broadly divided into Hodgkin’s lymphoma (HL) and non-Hodgkin’s B cell lymphomas (NHL) [1]. Both Hodgkin’s lymphoma and B-cell non-Hodgkin’s lymphoma often occur during adolescence [2,3,4].

In Poland, a detailed assessment of the prevalence of various forms of cancer within each group (International Classification of Childhood Cancer, ICC) shows that lymphomas account for 27% of malignancies in the 15–19 age category and 22% in the 10–14 age group [5,6].

Both HL [7] and NHL have good cure rates in adolescents [8,9]. Despite promising treatment results, it is crucial in the medical care provided to these young patients to optimize not only the treatment of the cancer process itself, but also the broader activities of the medical team. The ever-evolving holistic approach to adolescents suffering from cancer should also take into account other variables that determine health, such as emotional state, pain intensity level, sleep quality and the process of accepting one’s current health situation. Understanding these challenges can guarantee further improvements in both short- and long-term therapeutic outcomes [10,11,12].

Medical care provided to oncological adolescent patients should necessarily embrace psychological aspects, taking into account special vulnerability of this group to the occurrence of anxiety disorders and depression [13]. Emotional instability in healthy adolescents is mostly a transient condition, although it can also last until full adulthood [14,15]. A diagnosis of a life-threatening illness during this developmental period can further disturb adolescents’ emotional functioning. Kosir et al. [11] emphasize that this is a group of patients which requires a particularly individualized approach due to the low effectiveness of universal group interventions. The authors point out the need for a prompt diagnosis of mental disorders and intervention even when the symptoms are only subclinical [11].

One of the factors to be considered, especially in adolescents with ongoing cancer processes, is the quality of sleep. Sleep and wakefulness patterns in this group are a complex combination of processes related to development and maturation, but also mechanisms for regulating the daily rhythm [16,17] and lifestyle [18]. For adolescents who are experiencing a life-threatening disease, the quality of sleep becomes a priority. Scientific reports have proven a link between reduced NK cell activity and sleep disorders (insomnia), indicating the vital importance of sleep for immune defense against cancer cells [19,20,21,22]. Sleep disorders may be directly related to the cancer processes, the burden of the treatment and the hospital environment [23,24], but a two-way relationship between sleep quality and emotional suffering (anxiety, depression, irritability) cannot be excluded [23,25,26].

An inherent element of oncological disease and its treatment is the problem of pain sensations. Soltani et al. [27] showed that there is a close relationship between pain and insomnia and the development of mental health disorders and problems in adolescents, despite the fact that the adolescents studied were not burdened with a life-threatening disease.

Addressing the determinants affecting the functioning of an adolescent with cancer, especially in the early stages of treatment, can increase their chances of survival. They can also be considered significant in the process of cancer acceptance. As Sutin [28] points out, acceptance of the disease, understood as a rational attitude to the disease and giving a new purpose to one’s life, is important for achieving significantly better therapeutic outcomes.

Therefore, it is justified to conduct further research in this area and to deepen the knowledge regarding the acceptance of the disease by adolescents suffering from leukemia and lymphoma during the first cycle of anticancer treatment, including potential determinants typical of this period of treatment. Therefore, the purpose of this study was to assess adolescents’ acceptance of lymphatic malignancy during the first cycle of chemotherapy, taking into account an assessment of pain intensity, anxiety, depression and irritability/aggression, as well as the quality of sleep.

## 2. Materials and Methods

### 2.1. Study Design

A cross-sectional study was conducted to assess adolescents’ acceptance of lymphatic malignancy during the first cycle of chemotherapy. It also included an assessment of pain intensity, anxiety, depression and irritability/aggression, as well as the quality of sleep. The STROBE checklist was used in the preparation of the study report [29].

In order to achieve the assumed goal of the study, the following research questions were formulated:

1. What is the level of acceptance of the disease reported by the surveyed adolescents in the first cycle of chemotherapy?
2. What is the level of pain intensity, anxiety, depression, aggression/anxiety and sleep quality by gender which can affect adolescents’ level of acceptance of illness?
3. What is the relationship between acceptance of illness and adolescents’ gender, as well as the variables studied?

### 2.2. Sample, Setting and Data Collection

The study was conducted among adolescents diagnosed with lymphatic cancer during the first cycle of chemotherapy, at the Clinic of Pediatric Oncology and Hematology at a university hospital in southern Poland. There were two stages in the recruitment procedure allowing adolescents to participate in the study.

Stage one: analysis of medical records to identify adolescents meeting the criteria of age, clinical diagnosis and the treatment method used as well as the treatment stage. The age criterion included adolescents of both sexes from the age of 14 years to 16 years and 12 months (under 17 years of age). Such a narrow age group was chosen because of similar features of psycho-emotional development and the school education system in Poland (high school). The clinical criterion included diagnosis of lymphoid malignancy: Hodgkin Lymphoma—HL or non-Hodgkin’s Lymphoma—NHL. All patients were in the first cycle of chemotherapeutic treatment and had not undergone radiation therapy.

Stage two: from the entire group selected in the first stage, the researchers chose the adolescents who met further inclusion criteria, such as a stable health status, no acute or chronic comorbidities, no traumatic experiences, psychiatric treatment or psychological support before the disease, as well as a stable family and school situation in the last 6 months before the diagnosis. After a two-stage recruitment process and obtaining consent for voluntary participation in the study from both the adolescents and their parents, the study began.

Before deciding to participate in the study, each adolescent and parent was provided with verbal and written information about the purpose of the study, its course and the possibility of withdrawing from the study at any stage without giving a reason. The patients and their parents were informed that neither refusal to participate in the study nor participation in it would affect the quality of medical care and treatment. The group of selected adolescents as well as their parents gave voluntary consent for the collected material to be used for publication. The authors of the study explained to the adolescents the meaning of the concept of “disease acceptance” and other potentially misleading terms.

Parents did not take part in the study and did not participate in completing the questionnaires.

A statistical analysis was eventually performed on fully completed questionnaires collected from 19 girls and 31 boys.

The authors of the survey ensured respondents’ anonymity, confidentiality of data and privacy. In order to prevent the identification of adolescents by any other people unrelated to the survey, all information provided by respondents was coded and non-numerical information was removed. The survey was conducted in accordance with the ethical principles of the Declaration of Helsinki. The course of the study was approved by the Research Ethics Committee of the Jagiellonian University—Collegium Medicum (No. 118. 0043. 1.193.2024).

### 2.3. Participants and Involvement

Adolescents’ parents or other caregivers were not directly involved in the study, and the leading sources for further analyses were medical records and the information collected from the adolescents who met the study group selection criteria.

### 2.4. Description of Research Tools

The following survey questionnaires and scales were used in the study:

‑ Self-designed questionnaire—regarding sociodemographic data: respondents’ age and gender, education, type of disease and its stage of treatment.
‑ The Hospital Anxiety and Depression Scale (HADS), developed by Zigmond and Snaith [30], in the Polish adaptation by Majkowicz et al. [31]. An assessment of the usefulness of the scale for a group of Polish adolescents was conducted by Mihalica and Pilecka [32]. The scale is a screening tool. For the purposes of the current study, each subscale was analyzed separately (HADS-A for Anxiety, HADS-D for Depression, HADS-I for Irritability), as well as in total (HADS-T). Statistical analyses for each subscale except the HADS-I included a breakdown by category: Low, Medium, High. An analysis of the respondents’ answers was performed in accordance with the instructions of the authors of the scale and the authors of the adaptation.
‑ Numeric Rating Scale (NRS)—used to assess the level of pain intensity, where 0 means no pain at all, while 10 means the worst pain imaginable.
‑ Athens Insomnia Scale (AIS) was applied to assess the quality of sleep. Its validation to Polish conditions was performed by Fornal-Pawlowska et al. [33]. The scale allows for quantitative measurement of insomnia symptoms. For each question, the respondent gives an answer by selecting one of the four possibilities, from 0 points to 3 points, where 0 means no sleep problem or good quality of wakefulness, and 3 means the greatest severity of the problem and poor quality of wakefulness. A score of 8 or more indicates a high probability of insomnia in the respondent [34].
‑ Acceptance of Illness Scale (AIS) *—an acceptance of illness questionnaire developed by B. J. Felton and T. A. Revensson [35]. The adaptation to Polish conditions was performed by Juczyński and Ogińska-Bulik [36]. The total score, from 8 to 40 points, is an overall measure of the degree of the acceptance of illness. The lower the score, the worse the acceptance. The higher the acceptance, the better the adaptation and less psychological discomfort. Based on the AIS scores, 3 levels of illness acceptance have been established: 8 to 18 points indicates low acceptance of illness; 19 to 29 points—medium acceptance and 30 to 40 points—high acceptance.

The survey instruments used to assess anxiety, depression and irritability (HADS-M), sleep quality (AIS), pain (NRS) and acceptance of illness (AIS) are publicly available tools that are easy to use and interpret, not only for psychologists, but also for other members of the treatment team, such as nurses or physicians. They can be used as screening tests for the initial diagnosis of alarming symptoms.

* Note: due to the identical abbreviations for the Insomnia Scale and the Illness Acceptance Scale in Section 3, the authors of the study used the abbreviation AIS only for the Illness Acceptance Scale.

### 2.5. Statistical Analysis

Descriptions of quantitative variables such as age, the HADS, Acceptance of Illness Scale and Athens Insomnia Scale were presented using mean (M) and standard deviation (SD) due to their near-normal distribution. Categorical variables were described using counts and percentages. The characteristics of the study sample were presented by gender, and the significance of differences between boys and girls was assessed using the chi-square test of independence or Fisher’s exact test for categorical variables and using Student’s *t*-test for independent samples for quantitative variables. The strength and direction of relationships between pairs of individual scales were verified using Spearman’s rank correlation analysis. To verify which factors are associated with the occurrence of sleep disorders and acceptance of illness, a multiple linear regression analysis (with a continuous dependent variable of Athens Insomnia Scale or Acceptance of Illness Scale) and logistic regression (with symptoms of insomnia as a dichotomous dependent variable) were performed, taking into account potential confounders such as gender and age. The results were presented by estimating the corresponding effect measure—OR (odds ratio) or unstandardized linear regression coefficient (β) and the associated confidence interval (95% CI). To avoid overfitting and multicollinearity, models including HADS domains as explanatory variables were constructed separately for anxiety, depression and HADS-T, and we did not include more than four independent variables simultaneously in any of the models. Analyses were performed in the R package version 4.04, a significance level of 0.05 was set and two-sided statistical tests were used.

## 3. Results

### 3.1. Characteristics of the Study Group by Gender

Nearly two thirds of the group consisted of males (62%), average age of participants was 15.6 years with no difference between sexes.

The mean score on the Illness Acceptance Scale was statistically significantly lower for girls than for boys (15.89 vs. 17.29, *p* = 0.025); however, after categorizing the scores, there were no significant differences between the sexes. Lack of acceptance of the disease was found in 78.9% (n = 15) of girls and 74.2% (n = 23) of boys.

HADS-A and HADS-I scores were statistically significantly higher on average in women compared to men (8.53 vs. 6.45, *p* = 0.036 and 3.16 vs. 2.19, 0.029, respectively). For easier interpretation and better reference to clinical practice, these continuous variables were classified into low, medium and high levels. A high score for anxiety was obtained in 42.1% (n = 8) of the girls and 16.1% (n = 5) of the boys (*p* = 0.012). Regardless of gender, the study group (*p* = 0.143) was dominated by respondents with a low or moderate score for depression level. A high score corresponding to the possibility of depressive disorders was obtained in 10.5% (n = 2) of the girls and 22.6% (n = 7) of the boys. Gender did not differentiate the incidence of irritability/aggression in the study group (*p* = 0.074), in which a high and moderate score was obtained in 68.4% (n = 13) of the girls and 38.7% (n = 12) of the boys.

**Figure 1:**
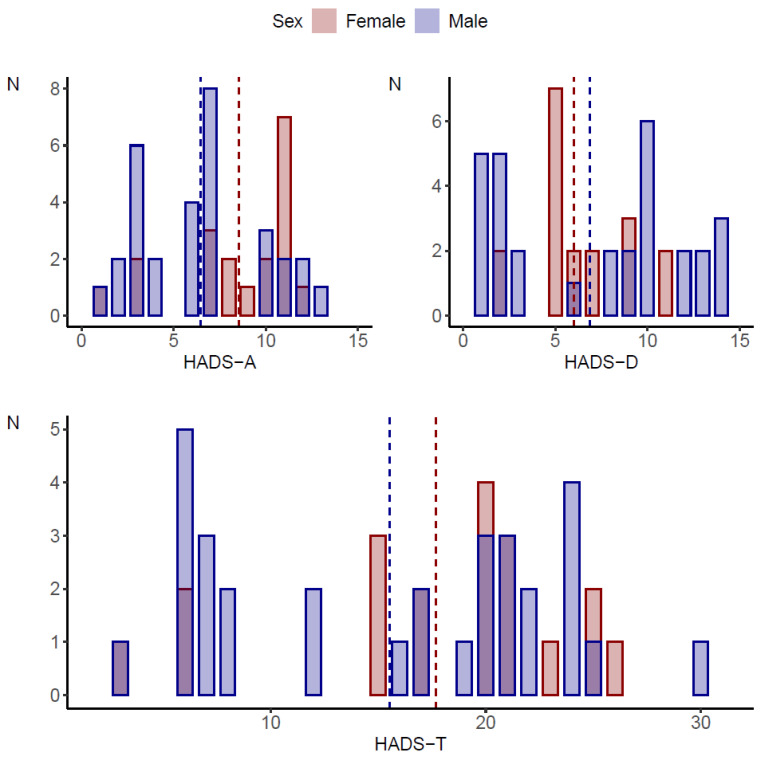
Comparison of HADS scale scores by gender of the subjects.

Sleep disorders were statistically significantly more common in girls (*p* < 0.001). On the other hand, the assessment of pain levels showed no statistically significant differences between girls and boys (*p* = 0.537). Details of the characteristics of the study group by gender are shown in Table 1 and Figure 1.

### 3.2. Analysis of Correlations Between HADS Scales, Illness Acceptance Scale, Insomnia Scale and Pain Levels

Except for the aggression domain, all HADS and HADS-T components correlated positively and statistically significantly with each other, and they were also positively correlated with insomnia and pain. The HADS-A, HADS-I and HADS-T were negatively correlated with acceptance of illness.

**Figure 2:**
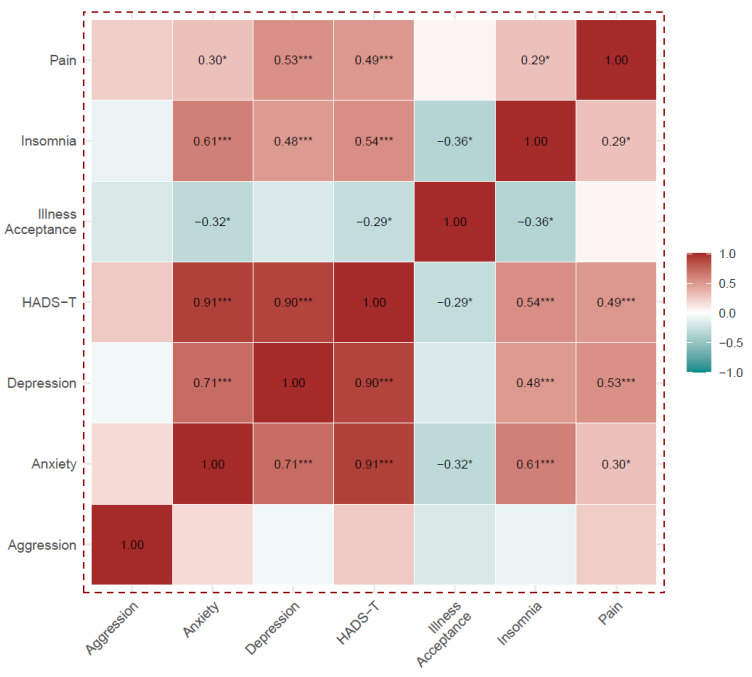
Analysis of Spearman’s rank correlations between the HADS scales, the Illness Acceptance Scale, the Insomnia Scale and pain level. Numerical values of correlation coefficients are presented only for correlations that were statistically significant, *** *p* < 0.001, * *p* < 0.05.

Acceptance of the disease correlated negatively with insomnia, but did not correlate with pain. Details are shown in Supplementary Table S1 and Figure 2.

### 3.3. Factors Associated with the Occurrence of Sleep Disorders in the Study Group

In order to fully illustrate the studied relationship between anxiety, depression and sleep quality, the HADS and insomnia scales were analyzed both as continuous and categorized variables. Regardless of the subjects’ gender and age, a 1-point increase on the anxiety, depression and HADS-T scales was associated with an increase of approximately 0.60, 0.49 and 0.26 points on the insomnia scale, respectively, and an increase in the chance of insomnia of 68%, 65% and 40%, respectively.

The mean score on the insomnia scale showed an increasing trend in the subsequent categories (Low, Medium, High) of each HADS scale. The mean values in the HADS-A, HADS-D and HADS-T subscales were higher by 6.70, 5.80 and 6.19 points for the High category, respectively, compared to the Low category.

An analysis of the HADS and Insomnia Scale as categorized variables showed significantly increased odds of insomnia for borderline conditions as well as anxiety and depression subscale disorders (Table 2).

The detailed results of the linear regression analysis verifying the association between the Athens Insomnia Scale and the HADS-A, HADS-D and HADS-T along with accompanying variables (age and sex) are given in Supplementary Table S2. Consistently, in all the analyzed models except the HADS-A considered as a categorized variable, sex was significantly associated with insomnia. Compared to girls, boys showed a lower mean insomnia scale score and a lower chance of insomnia. Age was not significantly associated with the score on the insomnia scale (Supplementary Table S2).

The relationship between pain and the quality of sleep was analyzed using linear regression. In the model adjusted for age and gender (Model 1) a 1-point increase in pain was associated with a 0.33-point increase in insomnia scale score. After additional standardization to any of the HADS subscales (Model 2–Model 4), the association of pain with insomnia levels was no longer statistically significant, while a 1-point increase in the HADS-A, HADS-D, HADS-T subscales was still associated with significantly higher insomnia levels (by a score average of 0.56, 0.51 and 0.26 points, respectively)—Table 3.

### 3.4. Study Variables vs. Acceptance of Illness

Because of the strong correlations between the HADS subscales and insomnia, their effects on disease acceptance were examined in four separate linear regression models, each with two variants: standardized to age only and standardized to age and gender. In the models standardized to age only (variant 1), higher levels on the insomnia, HADS-A and HADS-T scales were associated with lower disease acceptance (Model 1, Model 2 and Model 4, respectively).

When each of the four models was additionally standardized to gender (variant 2), none of the scales were statistically significant (*p* < 0.1). In the model with the HADS-T, the male gender had an average of 1.23 points higher disease acceptance compared to the female gender (Table 4).

## 4. Discussion

In the group of adolescents suffering from cancer, especially cancer involving hematopoietic and lymphatic systems, the treatment results and a good long-term prognosis are becoming better and better, although still not fully satisfactory [37]. Improving treatment efficacy is associated not only with modern drug treatment, but also with the need to optimize holistic medical care that takes into account patients’ psycho-emotional state. The benefits of the patient’s individual social involvement in the treatment process and attitudes toward their own disease [38] at every stage [39] are also emphasized. The diagnosis of the disease and the first cycle of treatment administered can be crucial to the acceptance of a difficult situation which the adolescents have to face, and can determine not only further therapeutic success [39,40], but also adolescents’ entry into adult life. Negative emotions associated with disease symptoms, diagnosis, the course of therapy and its side effects can have a destabilizing effect on a young person’s physical, social and psycho-emotional functioning [41,42,43,44], increasing the risk of suicide [41,45,46].

In the current study, almost 80% of girls and 75% of boys did not accept their illness, which may be due to the fact that adolescence is intrinsically characterized by the need for independence, separateness, self-realization and self-determination. The lack of satisfaction of these needs, resulting from the disease and its limitations, and the need to submit to the therapeutic process, may, especially in this group of patients, result in the occurrence of lack of acceptance, stress, irritability, rebellion, anxiety as well as fear of death [40,47,48]. These emotions, in turn, interfere with self-perception and the course of treatment regimens [49,50]. However, as Jin et al. [51] point out, there are no data on effective systemic identification and treatment of these problems. Although, in the present study, scores on the anxiety and irritability subscales and the HADS-T scale negatively correlated with the acceptance of the disease among adolescents, when the confounding variables of age and gender were introduced into the linear regression model, each scale lost its statistical significance. However, male gender remained a significant predictor of higher disease acceptance in the study group. Perhaps this was related to gender-differentiated coping strategies [52,53,54].

When facing illness, adolescents have to cope with the demands generated by sudden life changes. They need age-appropriate information and resources to confront new challenges [43]. Failure to meet these needs induces or exacerbates negative emotions among adolescents. In both the current study and reports by other authors, anxiety was the dominant emotion among adolescents [25,26,38,55], especially in girls. Also, high levels of irritability/aggression were observed, which is considered to be a frequently used method of expressing frustration [56]. In a meta-analysis presented by Osmani et al. [55], it is noted that adolescents suffering from cancer are more likely to experience emotional disorders, primarily anxiety, compared to older patients and healthy adolescents. Unfortunately, the authors of the cited study did not analyze emotion levels by gender.

In our study, mostly low to moderate levels of depression were observed in adolescents at the first stage of treatment, which is consistent with the results of Phan et al. [38], despite the application of different study tools. It cannot be ruled out that, at subsequent stages of treatment, with the prolonged duration of hospitalization, the level of depression in adolescents may rise. It is also indicated that the higher the level of depression, the lower the level of acceptance and adjustment to the disease [38]. Bearing this in mind, the study of the cancer adolescents’ emotional state should be included in the course of the entire therapeutic process and should be performed/implemented regularly, especially since depression in this group may have the characteristic of masked depression, which can only be recognized by observation of the patient’s behavior and their variable somatic complaints [57]. It is particularly in chronic diseases, including cancer, that the changing and inconsistent clinical picture is in contrast to the intensity of a person’s emotional and spiritual experiences [58]. In depression, pathological sadness co-occurs with impaired cognitive function, thought processes, will and decision-making abilities. In adolescents, depression, usually associated with anxiety, generates a sense of general helplessness and threat of death, especially in the face of severe somatic illness, which can also lead to contemplation of guilt and suicidal ideation [58]. It is noteworthy that male gender is an important determinant of suicide in adolescents with cancer, especially in the first 5 years after diagnosis [59]. Thus, it is necessary to take these issues into account in planning holistic patient care, keeping in mind the dynamics of the emotional state and its multidirectional determinants. What should be emphasized is the importance of programs that can prepare sick adolescents not only to receive knowledge about their health situation and manage their symptoms but also allow them to reduce anxiety and depressive symptoms and improve social interaction [39].

In the current study, gender as an independent variable did not determine pain intensity levels, similarly to the report by Harper et al. and Duran et al. [60,61]. However, the mediating role of gender in assessing pain levels in adolescents with cancer was indicated by Nunes et al. [62], according to whom higher pain levels in girls were associated with lower quality of life, which was not observed in the case of boys.

With increasing overall HADS scale scores, and increasing scores for depression and anxiety, higher levels of pain intensity and insomnia were observed, which was significantly more common in girls. Other authors [63] emphasize the multifactorial nature of sleep disorders in adolescents, as well as the links between various predictors, including biological factors, social factors, environmental factors, psycho-behavioral characteristics and psychiatric disorders, regardless of the level of the pain present [62]. Walker et al. [23], conducting a study in a group of adolescents with various types of malignancies, including leukemias and lymphomas, showed that adolescents receiving chemotherapy had significantly poorer sleep quality than their healthy peers; they also presented unfavorable sleep hygiene behaviors, and their daily rhythms were disrupted. Consequently, adolescents with sleep disorders may experience fatigue and distraction co-occurring with mood disorders, neurotic tendencies, and anxiety, resulting in a vicious cause-and-effect cycle [63].

Psycho-emotional and sleep disturbances at the initial stage of cancer treatment co-occur with functional impairment, which permanently contributes to a reduction in daily activities, self-care capabilities and the realization of developmental needs [64]. The disease and its consequences also lead to a sense of reduced autonomy [42]. It cannot be ruled out that these factors, too, may affect the level of acceptance of illness in adolescents. Thus, it would be beneficial to take this aspect into account when planning further research in this area.

### 4.1. Implications

Adolescent cancer, undoubtedly, disrupts the individual developmental trajectory. It is therefore necessary to personalize the assessment of the emotional state and level of disease acceptance at each stage, especially in the early stages of the therapeutic process. According to the young people surveyed, the research tools employed in the study were easy to use, filling out the questionnaire was not problematic and they “were not tiring” in the respondents’ opinion. In turn, for the researchers, they were easy to interpret and did not require the participation of a psychologist. The selection of these tools and the results obtained from the study may enable medical teams to develop optimal and individualized models of care for this group of patients. The medical team should be aware that, in subsequent stages of the disease and therapy, the patient will require different interventions than at the beginning, during the first cycle of chemotherapy. They should also be able to recognize the emotional situation of patients and their families and be sensitive to the multifactoriality of disorders and sources of stress including the dynamic course of adaptation to the disease. Taking these variables into account can help improve patient survival rates.

### 4.2. Limitations

The study was conducted at a single clinical center and included a small but highly homogeneous group of adolescents from different areas of Poland. Diagnostic and treatment protocols for oncological diseases are consistent across the country and in line with international standards. It seems that expanding the study to include other treatment centers would not affect the diversity of the study group, but would increase accessibility to patients, which could increase the precision of the estimated results and allow for standardization to more confounders, or stratification of the presented models by gender.

The study conducted was cross-sectional in nature. In planning future studies, it seems appropriate to include at least two time points of measurement in individual patients, which will allow for a more reliable analysis of the dynamics of emotion and acceptance of the disease and the development of a personalized care plan. Considering the fact that cancer treatment can change a teenager’s appearance, which is of great importance to this group of patients, the area of research should include the assessment of self-perception in the context of emotions. Also, taking into account the sense of purpose and meaning in life at different stages of struggling with cancer in future studies may be crucial to ensuring the psycho-emotional well-being of young patients.

The current study did not include an assessment of coping strategies, which may have determined both the level of emotions and the process of accepting the disease. Given the limitations presented, it is important to exercise caution in interpreting the results.

## 5. Conclusions

A significant group of patients did not accept their illness during the first cycle of chemotherapy. The predominant emotion observed among adolescents was anxiety, occurring primarily in girls. The level of depression in most subjects was low or moderate, and the level of irritability was high, regardless of gender. Sleep disorders were more common in girls and were associated with high levels of anxiety and depression. Regardless of gender, most adolescents experienced pain sensations. Scores obtained on the anxiety, irritability subscales and the HADS-T scale negatively correlated with the acceptance of illness among adolescents, but, when confounding variables were introduced into the linear regression model, each scale lost its statistical significance. However, male gender remained a significant predictor of higher acceptance of illness among the study group. Since the emotions of adolescents can fluctuate, the assessment of emotional states and the level of acceptance of one’s health situation in this group of patients should be implemented systemically, based on developed programs for detecting health problems with the possibility of adjusting them to individual needs. It is important that the implementation of this standard includes other members of the medical team, not just psychologists, which may be of the utmost importance especially in the case of developing countries, due to limited accessibility to specialists.

## Figures and Tables

**Table 1:** Characteristics of participants by gender groups.

Variable	Female	Male	*p*
	(N = 19)	(N = 31)	
Age, Mean (SD)	15.58 (1.12)	15.61 (1.17)	0.920
HADS-A, Mean (SD)	8.53 (3.2)	6.45 (3.36)	0.036
HADS-D, Mean (SD)	6 (2.92)	6.87 (4.9)	0.487
HADS-I, Mean (SD)	3.16 (1.68)	2.19 (1.33)	0.029
HADS-T, Mean (SD)	17.68 (6.54)	15.52 (7.75)	0.315
HADS-A categories, n (%)			
Low	6 (31.6%)	23 (74.2%)	0.012
Medium	5 (26.3%)	3 (9.7%)	
High	8 (42.1%)	5 (16.1%)	
HADS-D categories, n (%)			
Low	14 (73.7%)	14 (45.2%)	0.143
Medium	3 (15.8%)	10 (32.3%)	
High	2 (10.5%)	7 (22.6%)	
HADS-I categories, n (%)			
Low	6 (31.6%)	19 (61.3%)	0.074
Medium	5 (26.3%)	7 (22.6%)	
High	8 (42.1%)	5 (16.1%)	
HADS-T categories, n (%)			
Low	6 (31.6%)	14 (45.2%)	0.614
Medium	9 (47.4%)	11 (35.5%)	
High	4 (21.1%)	6 (19.4%)	
Insomia Scale, Mean (SD)	8.84 (1.46)	5.16 (3.9)	<0.001
Insomia Scale, n (%)			
No	4 (21.1%)	24 (77.4%)	<0.001
Yes	15 (78.9%)	7 (22.6%)	
NRS, Mean (SD)	6.21 (2.82)	5.9 (3.17)	0.730
NRS, n (%)			
Low (<3)	3 (15.8%)	9 (29.0%)	0.537
Medium	5 (26.3%)	8 (25.8%)	
High	11 (57.9%)	14 (45.2%)	
NRS			
Norm (<3)	3 (15.8%)	7 (22.6%)	0.827
Pain (≥3)	16 (84.2%)	24 (77.4%)	
Illness Acceptance Scale, Mean (SD)	15.89 (2.31)	17.29 (1.92)	0.025
Illness Acceptance Scale, n (%)			
No	15 (78.9%)	23 (74.2%)	0.967
Yes	4 (21.1%)	8 (25.8%)	

Note: *p*—*p*-value based on chi-square test of independence or Fisher exact test or Student *t*-test.

**Table 2:** Association between HADS-A, HADS-D and HADS-T (independent variable) and Athens Insomnia Scale (dependent variable)—multivariable (sex and age adjusted) linear and logistic regression analysis.

Variable	HADS		
	Anxiety	Depression	Total
Linear regression, with AIS as continuous dependent variable			
HADS as categorical			
Low, β [95% CI]	0.00 [REF.]	0.00 [REF.]	0.00 [REF.]
Medium, β [95% CI]	0.37 [−0.18, 0.92]	1.58 [−0.12, 3.28]	2.35 [0.89, 3.82] **
High, β [95% CI]	6.70 [4.87, 8.54] ***	5.80 [3.84, 7.76] ***	6.19 [4.38, 7.99] ***
HADS as continuous			
Per 1 point, β [95% CI]	0.60 [0.36, 0.84] ***	0.49 [0.32, 0.67] ***	0.26 [0.16, 0.37] ***
Logistic regression, with symptoms of insomnia as dichotomous dependent variable			
HADS as categorical			
Low, OR [95% CI]	1.00 [REF.]	1.00 [REF.]	1.00 [REF.]
Medium, OR [95% CI]	76.19 [3.61, 1609.56] **	15.71 [0.93, 266.59] *	32.77 [1.96, 548.13] *
High, OR [95% CI]	10.63 [1.99, 56.81] **	34.67 [1.86, 646.51] *	392.55 [12.65, 12,176.99] ***
HADS as continuous			
Per 1 point, OR [95% CI]	1.68 [1.24, 2.26] ***	1.65 [1.14, 2.39] **	1.40 [1.13, 1.72] **

All models of multivariable linear and logistic regression analysis were constructed separately for anxiety, depression and HADS-T (incorporated as independent variables) and were adjusted to sex and age; *** *p* < 0.001, ** *p* < 0.01, * *p* < 0.05.

**Table 3:** Linear regression analysis—relationship between pain incidence and the quality of sleep (Athens Insomnia Scale) in the study.

Model	Variables	β [95% CI]
Model 1	Sex, male vs. female	−3.58 [−5.37, −1.80] ***
	Age, per 1 year	0.12 [−0.65, 0.89]
	Pain, per 1 point	0.33 [0.04, 0.62] *
Model 2	Sex, male vs. female	−2.49 [−4.07, −0.91] **
	Age, per 1 year	0.58 [−0.10, 1.26]
	Pain, per 1 point	0.17 [−0.09, 0.43]
	HADS-A, per 1 point	0.56 [0.31, 0.80] ***
Model 3	Sex, male vs. female	−4.15 [−5.63, −2.67] ***
	Age, per 1 year	0.46 [−0.18, 1.11]
	Pain, per 1 point	−0.04 [−0.32, 0.24]
	HADS-D, per 1 point	0.51 [0.30, 0.71] ***
Model 4	Sex, male vs. female	−3.13 [−4.70, −1.57] ***
	Age, per 1 year	0.52 [−0.18, 1.21]
	Pain, per 1 point	0.04 [−0.25, 0.33]
	HADS-T, per 1 point	0.26 [0.13, 0.38] ***

Note: Results of the association with Insomnia Scale (as continuous) are presented via unstandardized linear regression analysis coefficients β with 95% CI. Model 1 includes sex, age and pain; Model 2: includes sex, age, pain and HADS-A; Model 3: includes sex, age, pain and HADS-D; Model 4: includes sex, age, pain and HADS-T; *** *p* < 0.001, ** *p* < 0.01, * *p* < 0.05.

**Table 4:** Linear regression analysis—relationship between acceptance of illness and HADS subscales and insomnia scale.

Variables	Variant 1 ^#^		Variant 2 ^##^	
	β [95% CI]	*p*	β [95% CI]	*p*
Model 1. Insomnia, per 1 point	−0.21 [−0.37, −0.06]	0.011	−0.16 [−0.34, 0.02]	0.090
Age, per 1 year	0.10 [−0.41, 0.60]	0.706	0.09 [−0.41, 0.59]	0.720
Sex, male vs. female	-	-	0.81 [−0.54, 2.15]	0.247
Model 2. HADS-A, per 1 point	−0.21 [−0.39, −0.03]	0.026	−0.16 [−0.35, 0.02]	0.095
Age, per 1 year	−0.10 [−0.64, 0.44]	0.724	−0.06 [−0.59, 0.47]	0.829
Sex, male vs. female	-	-	1.06 [−0.17, 2.29]	0.097
Model 3. HADS-D, per 1 point	−0.09 [−0.24, 0.06]	0.244	−0.11 [−0.25, 0.03]	0.141
Age, per 1 year	0.02 [−0.53, 0.57]	0.947	−0.01 [−0.53, 0.51]	0.979
Sex, male vs. female	-	-	1.49 [0.30, 2.68]	0.018
Model 4. HADS-T, per 1 point	−0.09 [−0.17, 0.00]	0.045	−0.08 [−0.16, 0.01]	0.078
Age, per 1 year	−0.07 [−0.61, 0.47]	0.797	−0.05 [−0.58, 0.47]	0.838
Sex, male vs. female	-	-	1.23 [0.05, 2.41]	0.046

Note: β—unstandardized linear regression analysis coefficient, ^#^ in Variant 1, models are adjusted to age, ^##^ in Variant 2, models are adjusted to age and sex.

## Data Availability

The data presented in this study are available on request from the corresponding author. The data are not publicly available due to specific ethical and privacy considerations.

## Supplementary Materials

The following supporting information can be downloaded at: https://www.mdpi.com/article/10.3390/healthcare13060637/s1, Table S1: Spearman’s rank correlation coefficients pairwise for HADS, Illness Acceptance Scale, Insomnia Scale, and pain levels. Table S2: Association between HADS and AIS (Athene Insomnia Scale)—multivariable linear regression analysis—full results for all incorporated variables, including covariates such as age and sex.
