# Supplementary material for: Acceptance of Illness, Quality of Sleep and Emotional State of Adolescents with Lymphatic Malignancy During the First Cycle of Anticancer Treatment—A Preliminary Report
Source: Healthcare (Basel). 2025 Mar 14;13(6):637. doi: 10.3390/healthcare13060637 (PMC11942510; doi:10.3390/healthcare13060637)
Supplement: Supplementary file 1 [file healthcare-13-00637-s001.zip › healthcare-3476424-supplementary.pdf]

Supplementary Table S1. Spearman's rank correlation coefficients pairwise for HADS, Illness Acceptance Scale, Insomnia Scale, and pain levels.

Scale	1	2	3	4	5	6	7
1. HADS-A							
2. HADS-D	0.705***						
3. HADS-I	0.177	-0.06					
4. HADS-T	0.913***	0.897***	0.256				
5. Insomnia	0.607***	0.483***	-0.101	0.542***			
6. Pain	0.296*	0.529***	0.237	0.494***	0.292*		
7. Illness Acceptance	-0.317*	-0.177	-0.187	-0.290*	-0.360*	0.051	

Note: *** $p < 0.001$, ** $p < 0.01$, * $p < 0.05$.

Supplementary Table S2. Association between HADS and AIS (*Athene Insomnia Scale)* – multivariable linear regression analysis – full results for all incorporated variables, including covariates such as age and sex.

**Variable**	**HADS**		
	**Anxiety, β [95% CI]**	**Depression, β [95% CI]**	**Total, β [95% CI]**
**HADS as categorical**			
Sex, male vs female	-0.29 [-9.10, 8.52]	-4.65 [-6.14, -3.16]***	-3.31 [-4.66, -1.96]***
Age, per 1 year	-1.58 [-2.97, -0.19]*	0.35 [-0.28, 0.98]	0.34 [-0.25, 0.92]
HADS-subscale, Medium vs Low	0.37 [-0.18, 0.92]	1.58 [-0.12, 3.28]	2.35 [0.89, 3.82]**
HADS-subscale, High vs Low	6.70 [4.87, 8.54]***	5.80 [3.84, 7.76]***	6.19 [4.38, 7.99]***
**HADS as continuous**			
Sex, male vs female	-2.45 [-4.04, -0.86]**	-4.13 [-5.58, -2.67]***	-3.13 [-4.67, -1.58]***
Age, per 1 year	0.58 [-0.11, 1.26]	0.46 [-0.18, 1.10]	0.52 [-0.16, 1.21]
HADS-subscale, per 1 point	0.60 [0.36, 0.84]***	0.49 [0.32, 0.67]***	0.26 [0.16, 0.37]***

Note: Results are presented via unstandardized regression coefficients β with 95% CI. Predictors such as anxiety, depression and HASD-T were incorporated as categorical or continuous variables, dependent on the model. *** $p < 0.001$, ** $p < 0.01$, * $p < 0.05$.
